# Developing and validating a measurement tool to self-report pedestrian safety-related behavior: The Pedestrian Behavior Questionnaire (PBQ)

**DOI:** 10.30476/beat.2020.86488

**Published:** 2020-10

**Authors:** Homayon Sadeghi Bazargan, Morteza Haghighi, Seyyed Taghi Heydari, Hamid Soori, Forouzan Rezapur Shahkolai, Seyed Abbas Motevalian, Reza Tabrizi, Minoo Mohammadkhani

**Affiliations:** 1 *Road Traffic Injury Research Center, Health Management and Safety Promotion Research Institute, Tabriz University of Medical Sciences, Tabriz, Iran*; 2 *Health Policy Research Center, Institute of Health, Shiraz University of Medical Sciences, Shiraz, Iran *; 3 *Safety Promotion and Injury Prevention Research Center, Student Research Committee, Shahid Beheshti University of Medical Sciences, Tehran, Iran*; 4 *Department of Public Health, School of Public Health & Research Center for Health Sciences, Hamadan University of Medical Sciences, Hamadan, Iran*; 5 *Department of Epidemiology, School of Public Health, Iran University of Medical Sciences, Tehran, Iran*

**Keywords:** Questionnaires, Traffic behavior scales, Pedestrians, Validity, Factor structure

## Abstract

**Objective:**

Pedestrians are road users vulnerable to traffic injuries and fatalities. This study aimed to develop and validate a pedestrian behavior questionnaire to be used in Iran.

**Methods:**

In this cross-sectional study, the initial questionnaire was designed based on the evaluation of previous studies conducted world-wide or in Iran. The initial pack included 127 items. After the assuring the face validity of the questionnaire, 27 experts’ opinions in the field of traffic was obtained for assessing/ improving the content validity. To test the reliability of the questionnaire, the test-retest method and internal consistency assessment were used. To evaluate the structural validity, the Exploratory Factor Analysis (EFA) using the principal component and the Varimax rotation was applied.

**Results:**

After completing the face validity and after summarizing the experts' suggestions, 12 questions were deleted. By calculating the content validity ratio and coefficient, 20 and 17 were removed. Also, the average content validity coefficient regarding relevancy, clarity and overall average were 0.86, 0.88, and 0.87, respectively. The Cronbach's alpha coefficient was 0.84. In the last stage and according to the results of the factor analysis, five factors violations, distraction, positive behaviors (group1), positive behaviors (group2) and Aggressive behaviors) were identified from the 29-items questionnaire, which explained 98% of the total variance.

**Conclusion:**

Considering the necessity of using a verified and validated tool for planning and evaluating effective interventions for pedestrians is inevitable. The tool designed in the study was found to be valid and reliable for use to measure pedestrian’s behavior and planning to modify high-risk behaviors and enhance safe pedestrian behaviors.

## Introduction

Pedestrian safety is a growing problem around the world. More than 270,000 pedestrians die on roads each year. According to the National Highway Traffic Safety Administration (NHTSA), there were 4,884 deaths of pedestrians and about 65,000, injuries caused by accidents in the United States during 2014 [1, 2]. It has been shown that Iran keeps to tolerate road traffic injuries as a major public health problem over the past two decades [3]. The findings show that road accidents in pedestrians are more than 30% in Iran and the problem is more prominent among the elderly [4, 5]. 

Although most pedestrians tend to use the opportunities created by the traffic to cross the street, passing over obstacles and diagonally are also common among pedestrians to save time or distance [6]. Among all road users, pedestrians have the most flexibility and can respond very quickly. On the other hand, they are often unpredictable and therefore increase the risk of road accidents [6-8]. In addition to traffic and environmental factors, in most cases of pedestrians and vehicle accidents, pedestrians’ vulnerability is associated with poor decisions or risky behaviors when crossing the road [9, 10]. The study of pedestrians behavior in 73 different signalized intersections in Nanjing, China, demonstrates the impact of factors such as safety, compliance with others, comfort, age, gender, length of the pathway and speed of vehicles [11].

Various studies in the world [8, 12-19] related to the provision of tools for measuring pedestrians’ traffic behavior and also the study of pedestrians’ traffic behaviors with questionnaire, observation and simulated environment showed that pedestrians’ attitudes and behaviors are effective in enhancing the risk of dangers. Wells *et al*. in a study showed that one-third of pedestrians showed insecure behaviors and distraction and the most common causes of unsafe behaviors and distraction were using headphones, texting and talking on cellphones, respectively [15]. In 2013, Garnia *et al*. developed a questionnaire containing 47 questions to examine the behavior of pedestrians of all ages in four factors: error, mistake, aggressive behavior and positive behavior. In this study, individuals aged between 35-45 years showed the highest positive behavior [17]. 

 In Iran, little has been done on developing a specific tool to cover all aspects of pedestrian safety behavior. In this regard, Hashemi Parast et al., [20] conducted a pilot study in Tehran based on the theory of planned behavior. Another study has been done by Pourdolat *et al*. developed a self-completion pedestrians’ red-light violation behavior questionnaire (PRVBQ) based on the theory of planned behavior [21]. Therefore, the research team decided to develop a tool with high validity and reliability in measuring the traffic behavior of pedestrians, considering the culture of Iran and according to the country's experts in the field of traffic in Iran.

## Materials and Methods

A psychometric study was conducted to develop a self-reporting tool for assessing pedestrian safety-related behaviors; The Pedestrian Behavior Questionnaire (PBQ). It was prepared in Persian and administered on pedestrian populations from four various districts including Tabriz, Shiraz, Hamden, and Tehran of Iran in 2018.

After developing the preliminary version of the questionnaire, different methods were used to iteratively determine and improve the validity and reliability of the tool and finalizing it into the application version. Several approaches were applied in order to ensure validity of the tool including face validity, content validity, and structural validity. For reliability, the test-retest reliability and internal consistency were assessed. Details of the steps towards developing the final questionnaire is provided as follows.

*Questionnaire design*

**Fig. 1:**
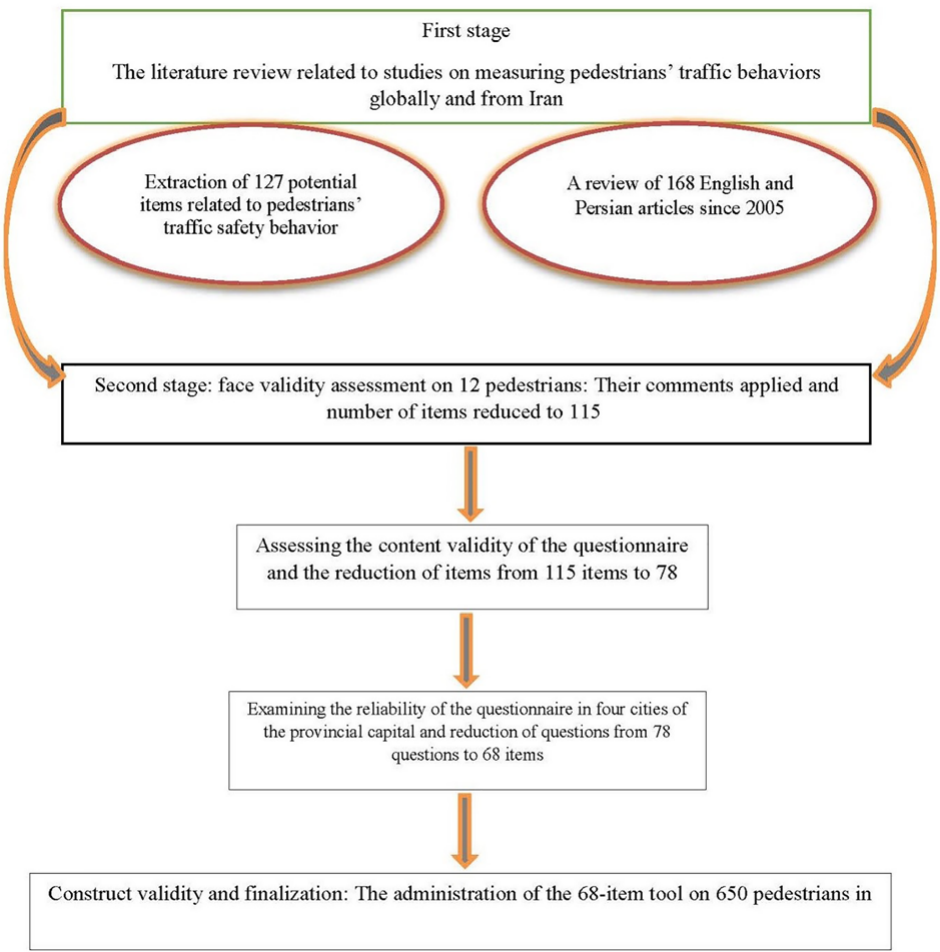
Process used for developing and assessment of the tool

To design the questionnaire, a literature review was initially conducted by the research team in association with studies on pedestrians’ traffic behaviors in Iran and other countries. After extracting the initial information through focus group discussions, a team of experts in field of road safety, health education, psychology and epidemiology developed a preliminary tool including 127 Likert-scaled items. This tool was evaluated for both content and face validity through expert views. Details of the process used for developing and assessment of the tool is provided in Figure 1.

*Face validity*

The face validity is the degree to which the appearance of the tool is suitable for collecting the information, especially from the viewpoint of the respondents [22]. To determine the face validity, the preliminary questionnaire was sent out to 11 experts in field of transportation management, road safety, epidemiology, health promotion, and psychology. Expert opinions were collected during a period of seven days. 

*Content validity*

The purpose of content validity is to ensure the ability of the tool to measure the phenomenon (concept) that it claims to measure [23]. All the items were sent out to 33 experts in various fields and they were asked to give their comments for each item in three aspects of relevance, clarity and necessity. They were asked to respond regarding each of the aspects of item assessment through a Likert scale of four choices including; "I totally agree"; "I agree" "I disagree"; and "I totally disagree". In case an expert disagreed with each item, he/she was given an opportunity in order to give explanations or improvement suggestions. Twenty-seven experts agreed to contribute who were provided face-to-face or by e-mail with a content validity assessment package. These experts were from a range of expertise in various fields of traffic/transportation management, law enforcement, road safety, epidemiology, education, health promotion and psychology. After improvement/reassessment iterations, the final statistics were reported and the questionnaire was considered for further processing. The content validity index and content validity ratios were calculated. Considering that the traditional methodology of assessing content validity index is a consensus-based routine, the authors applied the complementary the method proposed by Polit modifying the CVI using the Modified Kappa coefficient [22]. The minimum acceptable value for CVI based on this methodology is considered to be 0.78.

*Reliability*

After assessing the validity and preparing the 78-item questionnaire, the reliability of the questionnaire was determined using Cronbach's alpha (internal consistency) and the test-retest method. To this end, the updated version of PBQ was completed in 4 cities in North, west, south and central Iran (Tabriz, Shiraz, Hamden, and Tehran) by 178 people in two rounds with a two-week interval that 46 people from Tabriz, 53 from Shiraz, 41 from Tehran and 38 people from Hamedan participated in this part of the study which were purposefully selected according to cultural and social conditions from three points of each city. The agreement between the test and retest measurements was investigated. Intra-class Correlation Index (ICC), as well as Kendall's tau-b and Kappa Coefficients, were calculated.

*Construct validity*

After assessing the validity and reliability of the questionnaire and finalizing it, for measuring the structural validity at this stage, exploratory factor analysis was carried out using principal components extraction and Varimax rotation [24, 25]. For this stage of the study, 649 participants entered the study by cluster sampling. In this analysis, the KMO (Kaiser-Mayer-Olkin) statistic was used to examine the sampling adequacy. The output value of this index represents the amount of the input variance, and values above.6 represent the sampling adequacy. Moreover, the Bartlett's test of sphericity was used to examine whether the correlation matrix of the questionnaire items was not zero in the society [24].

*Statistical Analyses and Ethical Issues*

All EFA steps were performed using Stata statistical software package version 14 (Stata Corp. Texas). Ethical approval for this study was obtained from Research Ethics Committee in Shiraz University of Medical Sciences (IR.SUMS.REC.1396.S4).

## Results

The preliminary questionnaire, developed by the core expert panel, included 127 items. Through the face validity assessment process, 12 questions were removed from the preliminary questionnaire and the questionnaire with 115 questions was finalized at this stage. The reasons raised by the experts for removing the items included high similarity, ambiguity, and controversy with the aim of the study.

CVR and Modified CVI results indicated that all of the questions, except for 44 questions, had a score higher than 0.78 and therefore were recognized necessary and relevant. Out of these 44 items with a score lower than the index, 37 items were removed and seven items were re-examined by the research team due to their importance. After completing this step, the 115-item questionnaire was reduced to a 78-item tool to be assessed for reliability. Through assessing test-retest reliability, according to the ICC, Kendall's tau-b and kappa statistics, 10 items were removed due to their low ICC values. The test-retest reliability scores were obtained to be 0.77 for the whole questionnaire varying from 0.71 to 0.82 for its different subscales. The internal consistency was calculated to be 0.84 using Cronbach's alpha for PBQ. Finally, by removing 10 items due to low reliability, the tool was reduced to a questionnaire with 68 items to be entered into factor analysis step.

EFA was used for assessing the factor structure of the scale in order to 649 participants was invaluted. The factor analysis was found appropriate for identifying the factor structure of scale KMO and Bartlett's test of sphericity statistics were satisfactory for the developed factor model. Through an iterative process for EFA, the uniqueness of each item was examined and any item with a uniqueness score higher than 0.7 was removed after examining and comparing with other questions, which reduced the number of questions to 32 questions. Five factors were then extracted with Eigen values above one. In order to facilitate the interpretation and naming of the factors, 4 more items were deleted and 29 items formed the final questionnaire. The five factors comprised the subscales of the PBQ named as follows (Tables 1 and 2).

These factors could explain 98 % of the total scale variance. The first three factors could explain 76 % of the total variance. The internal consistency statistics of the final PBQ after EFA were satisfactory for the whole scale and all its subscales. The Cronbach's alpha for PBQ whole scale was 0.85. The lowest Cronbach’s alpha was calculated to be 0.700 belonging to Adhering to traffic rules subscale. 

**Table 1:** five factors comprised the subscales of the PBQ

**Subscale** **s**	**Behaviors **	**variance**	**Cronbach's alpha**
**Subscale ** **1**	positive behavior (groups 1)	0.412	0.873
**Subscale ** **2**	Violations	0.243	0.805
**Subscale ** **3**	positive behavior (groups 2)	0.171	0.762
**Subscale ** **4**	Distraction	0.095	0.828
**Subscale ** **5**	Aggressive	0.065	0.700

**Table 2:** The related factors and factor loads using principal component analysis method with Varimax rotation

**Question pedestrian’s behavior (PBQ)**	positive behavior (groups 1)	Violations	positive behavior (groups 2)	Distraction	Aggressive
For my convenience, I do not use pedestrian bridges, even if one of them is located near me.	0.54				
in order to save time, I pass the street or intersection indirectly or in a diagonal form	0.69				
I follow other people who pass the street unsafely in dangerous situations.	0.56				
If there is no suitable pavement, I try to walk in the direction of the vehicles.	0.67				
When I cross the street, I move spirally between vehicles.	0.58				
Because most people do not use pedestrian bridges, I do not use them either.	0.62				
To save time, I cross the street in a hurry and hastily.	0.51				
I pass the passage forbidden signpost, when I feel safe.	0.57				
I do not observe the pedestrians’ traffic rules because of non-observance of the rules by some traffic officers.	0.63				
When a person crosses the red-light unintentionally, I follow him/her regardless of the circumstance.	0.43				
I across the street, talking to a cell phone or listening to music with my headphones.		0.79			
Walking down the street or pavement, I use hands-free.		0.75			
I need to use cell phones even when walking and crossing the street in emergencies.		0.50			
When I cross the street, I use cell phone or text because I do not feel distracted.		0.71			
On a two-way street without crosswalk, I will pass through the first part and wait in the middle of the street to pass the second part if it is secure.			0.71		
When the truck or bus stops, I will pass through behind for my own safety.			0.69		
I use bright-colored or reflective clothes in the dark.			0.73		
I will cross the intersection after estimating the time of the vehicle arrival and the condition safety.			0.51		
In order to pass the street, I’ll not enter the street in a hurry and hastily.			0.47		
I cross the street after all the vehicles are stopped and the pedestrian light is green.			0.80		
Crossing the traffic, I stop in dangerous situations or I go back quickly.			0.72		
I move from the right side of the pavement so that I don’t bother others encountering me.				068	
I let the vehicles pass, even if the priority is to me.				0.62	
Crossing the street, I try to set an eye contact with the driver.				0.55	
Crossing the intersection, I observe the priorities.				0.66	
I follow the pedestrians’ traffic laws.				0.65	
In the twilight (the dark half at sunrise), I follow the pedestrians’ traffic laws more carefully.				0.60	
If I get angry with a driver’s behavior, I hit his/her car with hands or feet.					0.63
I get angry with other road users (driver, pedestrian, cyclist) and insult them					0.58

## Discussion

Around the world, pedestrian injury is a significant burden on health and is a major cause of death and disability, especially in the younger generation, and most of the pedestrians’ vulnerability results from poor decisions or their hazardous behaviors when crossing the road [9, 10]. The proportion of death among pedestrians in accidents in Iran is more than 22% of all crash fatalities the rate being higher in metropolises [26]. Since pedestrians are the most vulnerable road users, their safety has been the focus of transportation researchers living to development of pedestrian behavior assessment tools in several countries [17, 27-29]. Tools have also been developed in Iran for the assessment of some aspects of the pedestrians behavior but in a given area or city with regard to its cultural and geographical features [20]. To the best of our knowledge, no comprehensive questionnaire in Iran has been provided not only to take into account the cultural and social items in assessing pedestrian behavior, but also to include different areas of safety behavior such as cross-walking, red-light violations, and side walking in a comprehensive manner. Moreover, the available questionnaires have been developed based on data from very narrow populations considering geographical and cultural variations. In this study, we tried to design a questionnaire in line with the social and cultural characteristics of Iran. To this end, several provinces (with a mix of different cultures and geographical and climatic conditions) were involved through development and assessment of the validity and reliability of the expected instrument. 

Therefore, to provide the content of this questionnaire, the initial information with 127 items after extraction by the research team, was provided to experts in the areas of transportation and traffic safety, epidemiology, health education, psychology, and law enforcement who had a full recognition of the society and culture of the country. The validity of PBQ was confirmed both through face validity and content validity approaches. The reliability of the developed tool was confirmed both for internal consistency and test-retest reliability. The consistency among the items for the whole questionnaire was 0.77, varying from 0.71 to 0.82 for the five sub-scales of the questionnaire, which was within acceptable range. In a study by Shuchisnigdha Deb *et al*., [12] in the United States, this value was higher than 0.7 for the four subcategories of high-risk behaviors, except for positive behaviors.

After evaluating validity and reliability, 29 final items were classified into five domains: 1-Violations 2-distraction 3- positive behaviors (group1) 4- positive behaviors (group2) 5- Aggressive that extracted the components of domains Violations, Aggressive and Positive Behaviors are consistent with the results of the study by Sullman *et al*., [25, 17], Shuchisnigdha Deb *et al*., [12] in the United States, and the study by Granié M *et al*., [17] in France. As in Shuchisnigdha Deb *et al*., [12] in the United States, with a self-explanatory questionnaire, pedestrians’ behavior items were divided into five groups: violation of rules, errors, lapses, aggressive behavior, and positive behavior, in which the most reported item was positive behavior, followed by violations and infringement of the law. In study by Granié M *et al*., [17] in France, 20 items out of 47, were identified critical for the assessment of pedestrian behavior that were placed in four components as; violations, lapses, aggressive behavior, and positive behavior. Both in present study and the study by Granié M [30], positive behaviors were the most frequent pedestrian behaviors observed.

The five factors in this study were able to explain 98 % of the total variance of scale scores, of which the first three factors accounted for 76%. In the Antić B study, the five factors explained 66.4% of the total variance [31]. David Kaplan *et al*. is also stated that if the extracted factors explain 80% of the variance, the structural validity is acceptable [32]. In this study, the Cronbach's alpha coefficient for pedestrian behavior questionnaire was calculated to be 0.84 varying from 0.76 to 0.85 for each domain, which is a valuable and acceptable value [33]. In the Shuchisnigdha Deb study, Cronbach's alpha for domain positive behaviors was less than 0.6 [12] indicating a better internal consistency in our study. 

Compared to some of the most previous tools developed for measuring pedestrian behavior, an advantage of PBQ could be its parsimony with inclusion of only 29 items to cover five subscales [12, 17, 20]. This feature makes PBQ an easy to use tool in order to collect pedestrian behavior information even on sidewalks that there is a hesitancy to allocate time for participation in surveys. It is inevitable to use a valid and reliable tool for planning and evaluating the effective interventions regarding pedestrians. Therefore, PBQ, which has a relatively high validity and reliability, can be used for measuring pedestrian behavior and planning to modify the risky behaviors and enhancing the safe behaviors of pedestrians. No doubt, future use of the tool by other researchers’ other populations will be of help in improving the generalizability of PBQ. 

Due to the similarity of the items in two dimensions of positive behaviors (group 1 and 2), it was difficult for the research team to provide a better nomenclature in distinguishing the dimensions. For example, in group2, some positive behaviors are referred to as self-sacrifice, while in group1, majority of items refer to the adherence to laws and regulations. The authors hope that use of PBQ at larger samples and a wider cultural range of populations followed by performing CFA analysis may be helpful to address this issue.
